# Influence of 3D pore-scale heterogeneity on the physical, water transport, and mechanical properties of vesicular lavas from S. Miguel Island

**DOI:** 10.1038/s41598-026-47196-0

**Published:** 2026-04-03

**Authors:** Maria Luísa Pereira, Lucia Pappalardo, Gianmarco Buono, Alessia Falasconi, Nora Cueto, Rafael Fort, Carmen Vázquez-Calvo, Mário Moreira, Isabel Fernandes, Vittorio Zanon

**Affiliations:** 1 Instituto de Investigação em Vulcanologia e Avaliação de Riscos (IVAR), Universidade dos Açores, Rua Mãe de Deus, Ponta Delgada, 9500-321 Portugal; 2 Faculdade de Ciências e Tecnologia, Universidade dos Açores, Rua Mãe de Deus, Ponta, Delgada, 9500-321 Portugal; 3 Faculdade de Ciências, Instituto Dom Luiz (IDL), Universidade de Lisboa, Campo Grande, Lisboa, 1749-016 Portugal; 4 Istituto Nazionale di Geofisica e Vulcanologia (INGV), Sezione Osservatorio Vesuviano, Via Diocleziano, 328, Napoli, 80124 Italy; 5 Instituto de Geociencias (IGEO, CSIC-UCM), c/Doctor Severo Ochoa, 7, Madrid, 28040 Spain; 6 Departamento de Física, Instituto Superior de Engenharia de Lisboa, R. Conselheiro Emídio Navarro 1, Lisboa, 1959-007 Portugal

**Keywords:** Lavas, pore-scale heterogeneity, Digital rock physics, X-ray microtomography, Time-resolved tests, Capillary absorption, Energy science and technology, Solid Earth sciences

## Abstract

This study investigates how 3D microstructural variability influences vesicular lavas, which are globally widespread and important geo-resources. A lava block from S. Miguel Island (Azores, Portugal) was oriented along three orthogonal directions defined by pore elongation and analysed using laboratory methods. Mercury intrusion porosimetry and X-ray microtomography (µCT) characterised pore networks, and mechanical properties were measured through time-resolved in situ µCT tests. The lava sample has a heterogeneous, bimodal pore size distribution, comprising coalescent pores formed during magma ascent and lava emplacement, producing variability in 3D rock properties. Pore and throat size and heterogeneity distributions govern connected porosity and permeability across orientations, whereas directional variations in strength are influenced by the distribution of large, edge-proximal pores. Ultrasonic velocities and capillary absorption show limited directional variability at the mesoscale. Capillary water absorption is moderate and follows the Sharp Front model; pores larger than 1 mm contribute little to absorption, promoting predominantly gravity-driven flow despite low permeability. Our investigation demonstrates that strength variability in vesicular lavas is controlled by pore spatial distribution (rather than orientation alone), while large (> 1 mm) pores suppress directional capillary effects. This study also shows that similar multiscale approaches allow efficient exploration of pore-scale heterogeneity in volcanic rocks.

## Introduction

Heterogeneity, which describes the variation of properties with location within a material, is ubiquitous in lava rocks (i.e., lavas), owing to the variable processes involved in their origin and post-emplacement (e.g., Pereira et al.^1^. The effects of heterogeneity on physical, water transport, and mechanical properties extend across scales – from the microscale to the rock mass scale (e.g., Rodríguez-Losada et al.^2^; Pereira et al.^3^. Within this framework, laboratory analyses are often regarded as the primary stage for field-scale interpretations and are essential for improving the understanding and prediction of rock mass behaviour^4–6^.

Non-destructive techniques, such as water absorption via capillarity and ultrasonic wave velocities, serve as indirect proxies for the rock’s pore system. Complementarily, advanced methods such as X-ray microtomography (µCT) and mercury intrusion porosimetry (MIP), enable direct visualisation and quantification of the pore space system. Recent developments in µCT allow in situ mechanical testing during sample imaging, enabling the direct correlation between changes in rock microstructure and its mechanical properties (e.g., Pereira et al.^3^, Heap and Violay^7^.

Most studies investigating the influence of pores on rock properties employ numerical modelling^8,9^ or hybrid approaches combining experiments and simulations^4,6^. However, experimental validation remains essential to fully capture the variability of the pore space architecture of natural materials and better understand their performance. For instance, the pore size distribution (PSD) informs about the dynamics of bubble nucleation, growth, and coalescence^10^ during magma ascent and lava flow emplacement. These processes produce heterogeneous lava microstructures, which influence physical and geological properties^11,12^. Moreover, PSD governs water transport mechanisms and, consequently, the response of rocks when exposed to deleterious agents. In this context, capillary imbibition is a critical process for rocks exposed to atmospheric conditions^13,14^, with implications for volcanic slope stability and the degradation of building stones^15,16^.

The *P*- to *S*-wave velocity ratio (*V*_*p*_*/V*_*s*_), a common tool for seismic subsurface characterisation in volcanic settings^17^, is sensitive to the pore system^5^. Mechanical properties, influenced by the pore space, are also crucial for modelling volcanic behaviour and failure, as well as for the use of rocks in construction or as cultural heritage materials (e.g., Heap and Violay^7^,.

The present research investigates pore-scale heterogeneity and its influence on properties of vesicular lavas, a texture characterised by millimetric to centimetric pores representing fossilised gas bubbles trapped during cooling. The sample was collected on S. Miguel Island (Azores, Portugal), representing a lithology that is commonly used as aggregate and ornamental rock in the Azorean Archipelago.

The dry density (*ρ*) and connected porosity (*n*_*eff*_) of the cubic samples were examined. *P*-wave velocity (*V*_*p*_), *S*-wave velocity (*V*_*s*_), and capillary water absorption coefficient (*C*) were recorded along three orthogonal directions of the cubes. Because only three directions are assessed in this work, the term heterogeneity is used to describe property variations instead of anisotropy (directional dependence of properties), which requires measurements in more directions to be accurately defined. MIP allowed the determination of the mean pore radius (*r*), the mercury-intruded porosity (*n*_*eff_Hg*_), and the PSD, which were combined with the image-based porosity and absolute permeability (*k*) of 3D digital specimens analysed via µCT. Time-resolved mechanical tests enabled the determination of the unconfined compressive strength (*σ*_*c_UCS*_) and indirect tensile strength (*σ*_*t*_).

This study combines complementary methods to provide new multiscale insights into water transport and strength and their relationship with the pore system of the vesicular lavas. Particular attention is given to capillary processes, which are relevant in regions such as S. Miguel Island, where volcanic rocks are exposed to intense rainfall and conditions favourable to stagnant water^18^. In such settings, capillary and mechanical behaviour, together with permeability, may serve as indicators of rock collapse potential in both building stones and natural rock slopes^19,20^. Despite its importance, capillary water absorption remains comparatively underexplored in volcanic rocks^16,20–26^, especially when contrasted with the extensive literature on sedimentary lithologies and permeability-focused studies (e.g., Lavallée and Kendrick^27^. To address this gap, this work provides new experimental evidence and interpretations of capillary water absorption in vesicular lavas, linking pore-scale heterogeneity with their physical and mechanical behaviour.

## Materials and methods

### Sample collection

Fogo (or Água de Pau) Volcano is a ~ 200,000-year-old stratovolcano in central S. Miguel Island (Azores, Portugal) with a history of seismicity and recent unrest episodes^28,29^, composed of lava flows and pyroclastic deposits.

The fresh vesicular lava (BF35) was sampled from accessible outcrops in Ribeirinha Parish, where lava flows commonly exhibit vesicular textures with millimetric to centimetric pores formed by gas trapped in cooling magma. The BF35 basalt/andesite (s.l.) hand-specimens closely resemble the basaltic trachyandesite analysed by Pereira et al.^3^ and other lavas used as building materials on the island. Therefore, the sample represents a typical porous volcanic rock suitable for studying pore-scale heterogeneity.

**Fig. 1:**
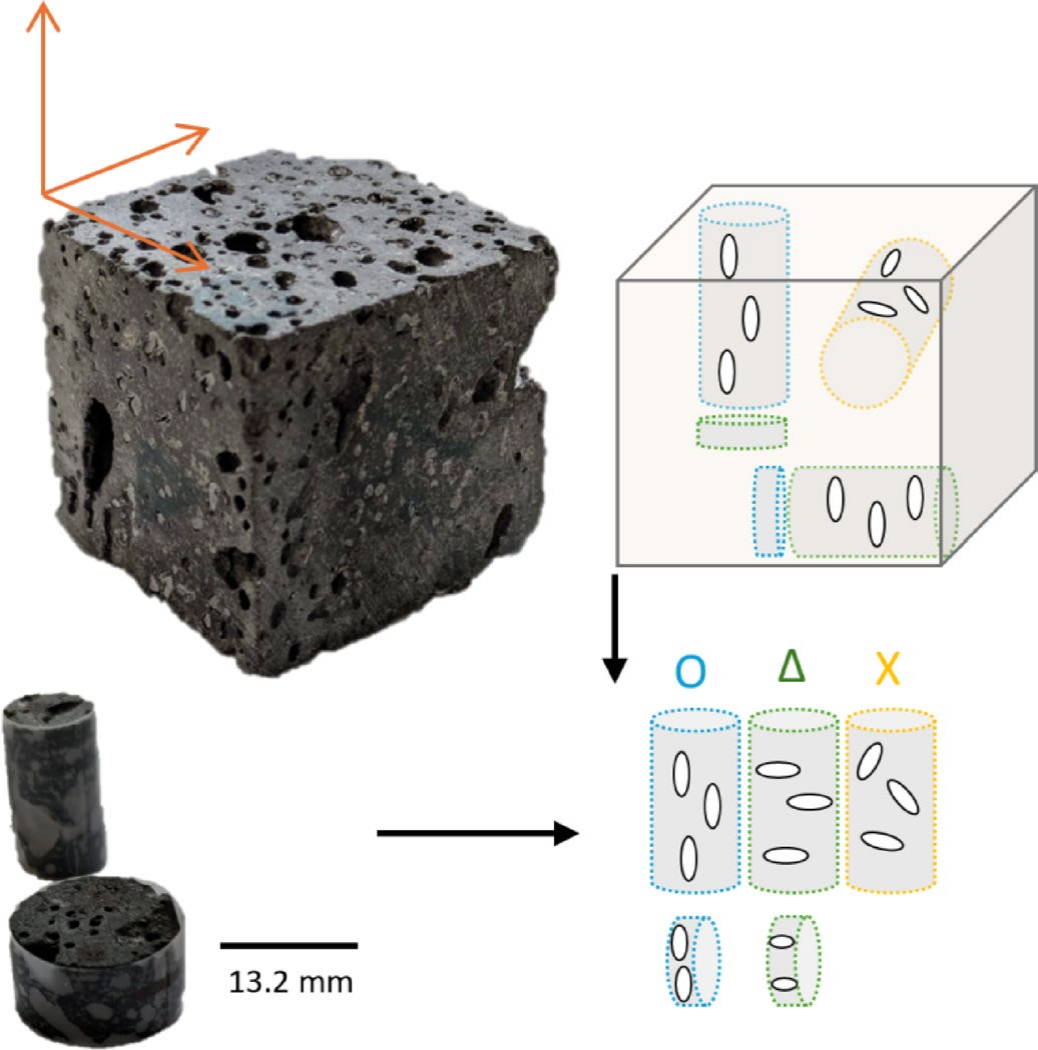
BF35 cube and directions defined by pores parallel (O), perpendicular (Δ), or mixed (X) for the measurement axis. Cylinders drilled cored along O, Δ, and X (BF35.1) and discs along O and Δ (BF35.2).

Six cubic specimens (28–31 mm) were prepared from BF35 (hereafter denoted BF35.1 to BF35.6), and further sub-sampled into cylinders, discs, and prisms. The cubes’ faces were visually organised considering the preferred orientation of pores to define three orthogonal directions relative to the measurement axis: parallel (O), perpendicular (Δ), or in between (X) (Fig. 1). This directional framework was strictly maintained during the drilling of the cylinders. For the discs, the drilled cores were inverted to align the pore preferred orientation with the loading axis (Fig. 1).

### Saturation and buoyancy technique

The cubic specimens were tested following the ISRM suggested method^30^. The saturation and buoyancy technique was used to obtain the connected porosity, *n*_*eff*_ (Eq. (1)), and the dry density, *ρ* (Eq. (2)), expressed in g/cm^3^. These properties were calculated regardless of specimen orientation because they are bulk properties.

The method involves measuring three masses (g) - dry mass (*M1*), submerged mass (*M2*), and saturated mass (*M3*). After oven-drying for 70 ± 5 °C for at least 48 h^31^, the saturation was achieved by immersing the cubes in distilled water under vacuum (800 Pa) in a sealed chamber for 24 h.

During the first hour, periodic agitation of the chamber facilitated the removal of air from the pore spaces, in accordance with ISRM^30^. After saturation, *M2* was measured by weighing the specimens while suspended in a tared basket in water. Finally, *M3* was recorded immediately after *M2* following the removal of surface water with a damp cloth. These masses were then used to estimate *n*_*eff*_ and *ρ* as follows:

1 $$\:{n}_{eff}\left(\%\right)=\:\frac{M3-M1}{M3-M2}\:\times\:\:100$$

2 $$\:\rho\:\:(g/c{m}^{3})=\frac{M1}{M3-M2}\:$$

### Ultrasonic wave velocity test

*P*-wave (*V*_*p*_) and *S*-wave (*V*_*s*_) velocities were determined using the through-transmission technique on dried cubes, with arrival times were measured along the three orthogonal directions. A Panametrics^®^ 5072 ultrasonic pulse generated the signals, operating at 2 kHz with energy levels ranging from 20 µJ to 50 µJ, adjusted according to signal attenuation. Signal acquisition was carried out using a Tektronix^®^ TDS 210 oscilloscope.

Different transducer types and operating frequencies were employed for *V*_*p*_ and *V*_*s*_ measurements. *P*-waves were recorded using Physical Acoustics R6-alpha sensors (frequency range: 35–100 kHz), while *S*-waves were recorded using SH10 sensors (frequency range: 100–200 kHz) from the same manufacturer.

Arrival times were manually picked from the digital oscilloscope, and wave velocities were calculated by dividing the distance between the transducers by the signal propagation time. The estimated error in the distance measurement is 0.2 × 10^− 3^ m, whereas the error in determining the signal arrival time by visual observation is 0.1 × 10^− 6^ s. The final estimated error for the average velocities is lower than 3%.

Dynamic Young’s modulus *E*_*d*_ (Eq. (3)) and Poisson’s ratio *υ*_*d*_ (Eq. (4)) were calculated following ASTM D2845-08^32^.

3 $$\:{E}_{d}\left(Pa\right)=\:\left[\rho\:{{V}_{s}}^{2}\left(3{{V}_{p}}^{2}-4{{V}_{s}}^{2}\right)\right]/({{V}_{p}}^{2}-{{V}_{s}}^{2})$$

4 $$\:{\upsilon\:}_{d}=({{V}_{p}}^{2}-2{{V}_{s}}^{2})/\left[2({{V}_{p}}^{2}-{{V}_{s}}^{2})\right]\:$$

### Capillary absorption test

The capillary water absorption test was conducted according to the NP-EN 1925:2000 standard^31^, along the three orthogonal directions of each cubic specimen. Cubes (with sides of ~ 30 mm) were oven-dried at 70 ± 5 °C for at least 48 h, cooled in a desiccator, and weighed before each test cycle. The specimens were then placed on supports and partially immersed (3 ± 1 mm depth) in a water tank, which was covered to prevent water evaporation.

Mass gain was recorded at standard-specified intervals – 1, 4, 10, 15, 30, 60, 480, and 1440 min. Water absorption at each time step (*i*) was calculated as the mass per unit area, *Y(i)*:

5 $$\:Y\left(i\right)\:\left(g/{m}^{2}\right)=\:a\times\:b\sqrt{t\left(i\right)}$$

where *a* is the maximum absorbed water per exposed surface area (g/m^2^) and *b* is the inverse square root of time (s^0.5^), following the Annexe of the standard^31^.

The standard water absorption coefficient (C_*EN*_; g/m^2^·s^0.5^) was derived as the product of parameters *a* and *b*^31^, whereas the capillary connected porosity (*n*_*cc*_) value was determined using Eq. (6):

6 $$\:{n}_{cc}\left(\%\right)=\:\frac{(Mf-Mi)}{V\:\times\:{\:\rho\:}_{l}}\:\times\:100\:000$$

where *Mi* (g) is the initial dry mass, *Mf* (g) is the final saturated mass, *V* is the specimen volume (mm^3^), and *ρ*_*l*_ is the liquid density of water at 25 °C (0.997 g/cm^3^)^33^.

Sharp Front (SF) model^34–36^ was also considered to analyse capillary curves. In this model, water absorption is the sum of the two absorption processes: *C*_*e*_ = *C*_*a*_ + *C*_*m*_, where *C*_*e*_ represents the initial rapid capillary absorption along coarser, well-connected pores; *C*_*a*_ is the intermediate anomalous coefficient describing a transient stage where the contribution of larger pores decreases while that of finer pores increases; and *C*_*m*_ corresponds to the late-stage slow absorption through fine pores, increasingly influenced by gravity. In the capillary curves, *C*_*e*_ and *C*_*m*_ correspond to the slopes of the initial and late linear t^1/2^ kinetics, whereas *C*_*a*_ represents the deviation from this linear trend.

### Mercury intrusion porosimetry

A prismatic specimen (7 × 7 × 17 mm) was cut from BF35.6 cube without considering pore orientation and oven-dried at 70 °C until constant mass prior to testing.

The MIP analysis was performed using a Micromeritics^®^ AutoPore IV 9520 porosimeter, covering a pore radius range from 0.0015 to 180 μm. Penetrometers with a maximum measurable intrusion volume of 0.366 cm^3^ and a total stem volume of 0.392 cm^3^ were designed for solids with a five cm^3^ sample volume.

During the test, increasing pressure (up to 414 MPa) forced mercury progressively into the pores from largest to smallest. Intrusion and extrusion volumes were recorded at each pressure step. The PSD reflects the incremental intrusion volume, whereas the cumulative curve represents the cumulative intrusion as a function of pore radius.

### X-ray microtomography and time-resolved mechanical tests

Three cylinders (7.7 mm diameter; length-to-diameter ratio of 2) were drilled from cube BF35.1 along the X, O, and Δ directions, while two discs (13.2 mm diameter; thickness-to-diameter ratio of 0.6) were extracted from cube BF35.2 along O and Δ (Fig. 1).

Time-resolved mechanical tests were conducted using a Deben in situ load cell stage (CT5000H250; 5210 N capacity; validated in^37^ integrated within a ZEISS^®^ Xradia Versa-410 µCT system. Cylinders were scanned at 90 kV, 110 µA, and 0.4× magnification, acquiring 801 projections over a 180° rotation to yield a voxel resolution of ~ 15 μm.

Scans were systematically performed before and after uniaxial compressive strength testing (UCS). For Brazilian (indirect tensile strength; ITS) tests, real-time µCT imaging was acquired using the same settings to monitor crack propagation during brittle failure. A constant displacement rate of 0.03 mm/min was maintained.

The uniaxial compressive strength *σ*_*c_UCS*_ and the indirect tensile strength *σ*_*t*_ were determined as follows^30^:

7 $$\:{\sigma\:}_{c\_UCS}\left(MPa\right)=\:\frac{P}{\:A}$$

8 $$\:{\sigma\:}_{t}\left(MPa\right)=\:\frac{2\pi\:P}{\:Dt}$$

where *P* is the peak load (N), *A* the surface area (mm^2^) of the cylinder, *D* and *t* are the respective diameter (mm) and thickness (mm) of the discs.

Core specimens scanned via µCT (pre- and post-UCS test) underwent digital rock physics analysis using PerGeos^®^ software. Dedicated modules were used to segment and quantify 3D pore features. Image segmentation was initially performed using the software’s automatic thresholding algorithm (Otsu criterion using Auto Thresholding module), followed by manual validation and minor refinement (through Interactive Overlay Threshold module) to ensure accurate phase segmentation. The resulting segmentations (automatic vs. manual) produced negligible differences in computed pore metrics, confirming the robustness of the procedure.

Quantified parameters included total (*n*_*T*_; %) and connected porosity (*n*_*eff*_; %), centroid path tortuosity (*Ƭ*, dimensionless), absolute permeability (*k*, m^2^) via the Lattice Boltzmann method, as well as pore aspect ratio (major-to-minor axis ratio), equivalent radius (µm), and inclination (dip angle, °) relative to the horizontal of the specimen, ranging from perpendicular (0°) to parallel (90°) to the loading direction. Moreover, feldspars (i.e., the main mineralogical phase) were also segmented to estimate their volume fraction.

A filter analysis was performed prior to pore space metric extraction to remove objects smaller than two voxels, thereby reducing uncertainty due to noise^38,39^.

Pore network models (PNM) were developed for the UCS pre-deformed cylinders using a dedicated module in PerGeos^®^^40^. In this model, pores (spheres with equal pore volumes) are interconnected by throats (cylindrical connections between pores), through a specified number of connections represented by the coordination number (CN).

To enable comparison of pore characteristics across the different drilled cylinders, the mean equivalent pore radius and the pore volume were computed for each cylinder and dip interval (10°). For each parameter, a dimensionless fraction was obtained by normalising the mean equivalent radius and the pore volume, independently, with respect to their respective global sums across all cylinders and dip intervals. These fractions quantify the relative contribution of each measurement to the overall pore radius and pore volume distributions.

To emphasise the influence of larger pores, the radius fraction and the volume fraction were multiplied by their corresponding equivalent radius and pore volume, yielding a radius-weighted equivalent radius and a volume-weighted pore metric, respectively. These weighted parameters were used to establish separate ranking systems for pore radius and pore volume, in which higher values correspond to larger pores.

Finally, a combined pore size ranking system was defined as the product of the radius-weighted equivalent radius and the volume-weighted pore metric. By assigning equal weight to pore radius and pore volume, this index provides an integrated ranking that reflects their joint contribution to the pore structure.

## Results

### Pore space characterisation

#### MIP analysis

**Fig. 2:**
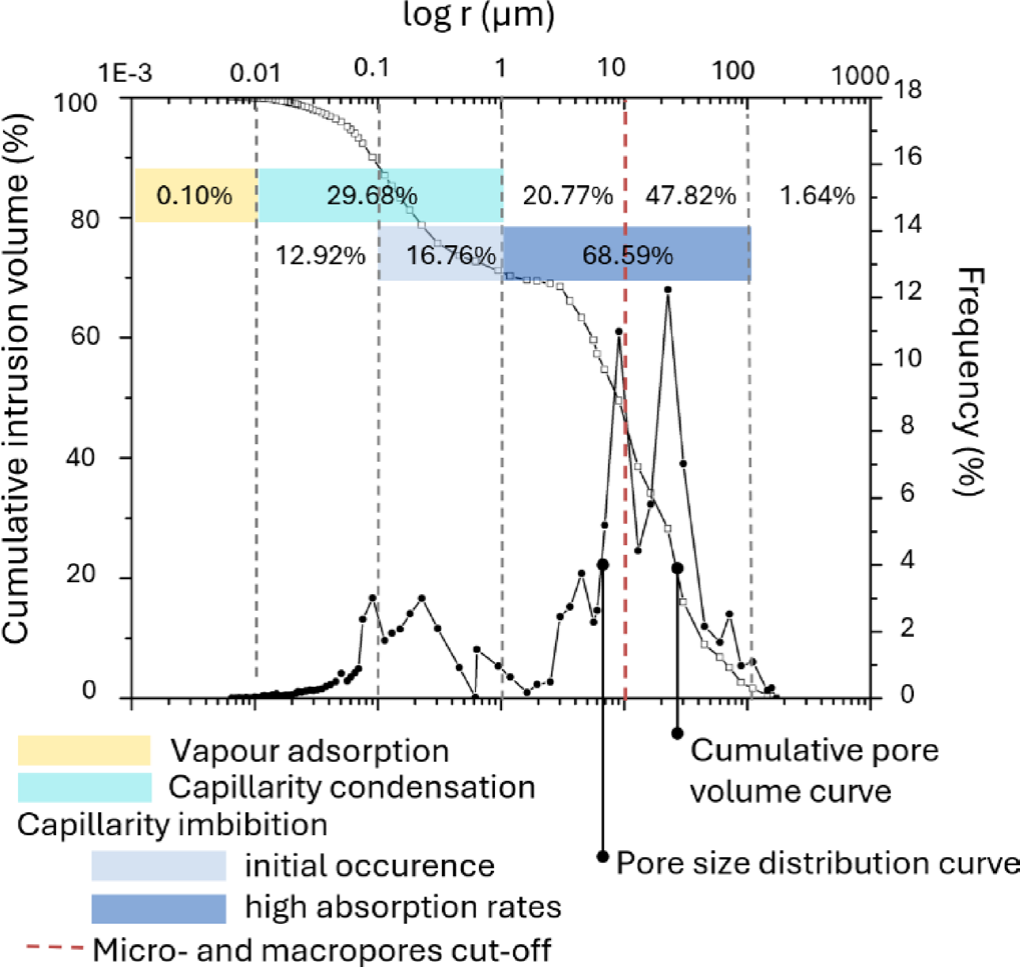
Cumulative mercury intrusion (open squares; cumulative intrusion volume vs. pore size) and pore size distribution (solid circles; pore size vs. frequency) curves of BF35, showing a non-uniform stair-shaped trend and a bimodal pore size distribution, respectively. Pore radii are related to water transport mechanisms (vapour adsorption, capillarity condensation, and capillarity imbibition) following the colour scheme. The volume of pores is represented for the several pore radii interval (µm) in %.

MIP enabled the analysis of pores with radius from 0.0015 to 180 μm, providing the PSD and mean radii *r* illustrated in Fig. 2. A 15 μm cut-off was used to distinguish micro- from macropores, following the approach of Pereira et al.^3^ and Franzson et al.^41^. Results on the cumulative intrusion volume show that BF35 contains 35.1% of macropores (pore radius > 15 μm) combined with *r* = 0.278 μm and a *n*_*eff_Hg*_ = 12.81%.

PSD plot of BF35 shows a bimodal distribution, with 68.29% of the pores having radii of 1–100 μm. The cumulative pore volume curve, following the describing schemes of Tuğrul^42^, Dinçer and Bostanci^25^, and Pereira et al.^3^, is non-uniform with an evident stair shape (Fig. 2).

The coloured intervals of Fig. 2, representing the dominant water transport mechanisms^13,42^, indicate that BF35 has a significant number of pores within the size suitable for active capillary imbibition (0.1 to 100 μm). However, hand-sample inspection confirms that many pores in BF35 exceed the detection limit of MIP analysis. As a result, MIP results were complemented by digital rock analysis.

#### Digital rock physics

##### Pore network characteristics

**Fig. 3:**
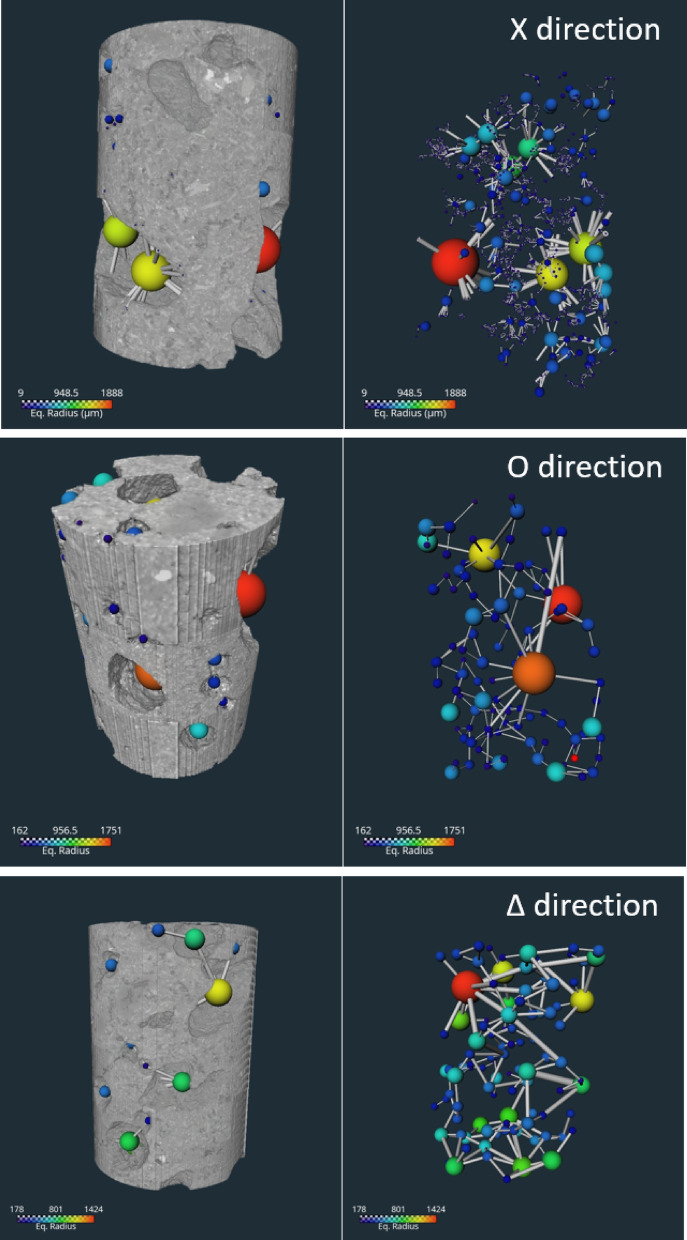
Pore network model (PNM) of BF35.1 specimen for the cores produced along X, O, and Δ directions.

**Fig. 4:**
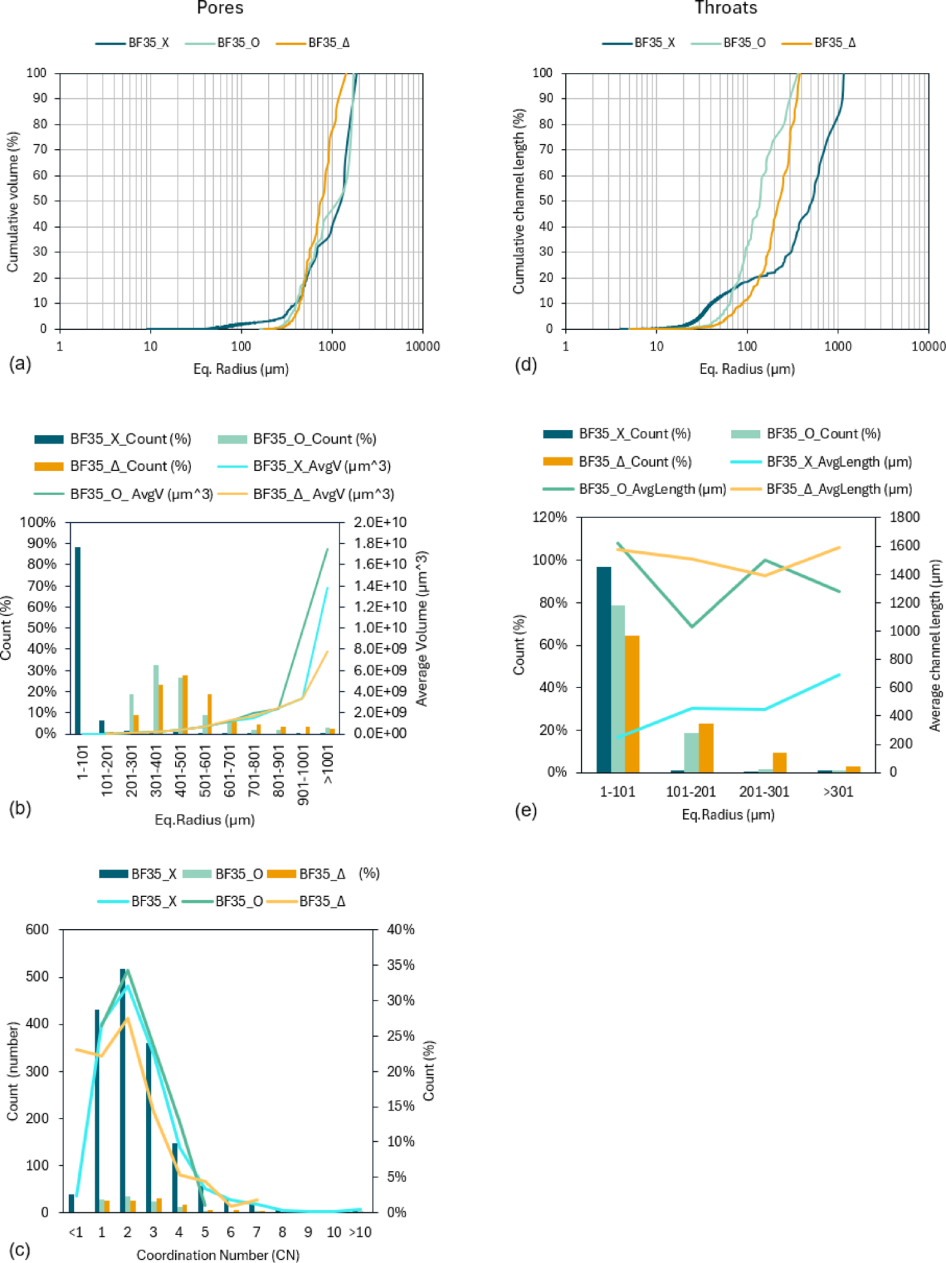
Quantitative results of the pore network models produced in the X, Δ, and O cylinders of BF35. All pores are larger than two voxels (i.e., > 30 μm). Information regarding pores (spheres with equal pore volumes) is presented in: (a) cumulative mean volume along equivalent radius of the pores; (b) number of pores per each group of equivalent radii; and (c) coordination number and its number (bars) and percentage to the total (lines). Information on the throats (cylindrical connections between pores) is present in: (d) cumulative mean channel length along equivalent radius of the throats; (e) number of throats per each group of equivalent radii.

The results of image analysis of µCT scans, obtained at a resolution of 15 μm, are summarised in Table 1. Each digital cylinder is further characterised by a PNM (Fig. 3), where pores are scaled based on their equivalent radius and throat thickness represents the channel length. Furthermore, the quantification of pores (Fig. 4a-c) and throats (Fig. 4.d, e) complement MIP analysis, allowing a more complete pore-scale characterisation.

**Table 1:** Physical, water transport, and mechanical properties of the vesicular lava BF35 in the X, Δ, and O direction. *n*_*T*_ – total porosity; *n*_*eff*_ – connected porosity *k* – absolute permeability; *Ƭ* – tortuosity; *Fp* – feldspar content; *σ*_*c_UCS*_ – unconfined compressive strength; *σ*_*t*_ – indirect tensile strength.

Sample	Cylinders										Discs
	Pre-deformation (before test)					Post-deformation (after failure)					
	*n*_T_ (%)	*n*_eff_ (%)	k (mD)	T	Fp (%)	*n*_T_ (%)	*n*_eff_ (%)	k (mD)	T	σ_c_UCS_ (MPa)	σ_t_ (MPa)
BF35_X	24.52	18.61	6.63	1.8	16.00	25.75	22.65	30.42	1.8	35.99	-
BF35_O	23.38	20.06	1.68	1.9	19.40	24.67	23.11	31.54	1.7	15.45	3.89
BF35_Δ	22.68	20.66	0.66	2.1	18.88	24.02	22.62	4.47	1.8	32.46	1.97

The cored cylinders show heterogeneous pore structures at the microscale. *n*_*T*_, along the three directions, ranges between 22.68 and 24.52% and is inversely proportional to *n*_*eff*_ (18.61–20.66%) and *T* (1.8–2.1), and directly proportional to *k* (0.66–1.8 mD). These values show no apparent correlation with the feldspar content (16–20%), the main mineralogical phase, although a general decrease in *n*_*T*_ with increasing crystallinity can be observed.

Overall, the core extracted along the X direction (i.e., in between parallel and perpendicular to the preferred orientation of pores), followed by BF35_O, shows the most heterogenous distribution of pores and throats at the microscale, containing the highest number of smaller pores (equivalent radius < 950 μm; blue in Fig. 3).

Figure 4b supports this observation, with BF35_X exhibiting a higher pore frequency clustered within the 1–101 μm range. The cumulative throat channel length curve for BF35_X (Fig. 4d) covers a broader distribution (stair-step pattern), with most throats ranging from 20 to 1000 μm. However, the large number of short throats (Figs. 3 and 4e) leads to the lowest mean channel length (Fig. 4e).

All three digital cores contain large pores (in yellow to red in Figs. 3 and 4b). These correspond to coalescent pores (i.e., often comprising multiple spheres in the de-coalesced PNM), usually connected by longer (and thicker in Fig. 3) throats. BF35_X, like BF35_O, has a significant proportion of pores with radii greater than 100 μm, with some exceeding 1 mm (Fig. 4a). Although the coordination number (CN; Fig. 4c) shows that most pores have one to three connections in all cylinders, the presence of numerous pores with CN > 2 in BF35_X, some exceeding seven connections, may explain the lowest measured tortuosity, *Ƭ*, and highest permeability, *k*, despite this cylinder having the lowest *n*_*eff*_.

The Δ and O direction cores (i.e., perpendicular and parallel to the preferred orientation of pores, respectively) display a more open pore structure comprising large pores predominantly located near the core boundaries (Fig. 3), combined with relatively uniform cumulative throat channel length curves (Fig. 4d). These cores have a significant pore frequency in the 201–601 μm interval (Fig. 4b), while throat radii are mainly between 50 and 120 μm (Fig. 4e).

Particularly for BF35_Δ, the steep cumulative pore volume curve dominated by pore radii between 100 and 1100 μm (Fig. 4a) indicates a more uniform pore size distribution, reaching the lowest mean volume (Fig. 4b). The throats of this cylinder have similar length (Fig. 4e) and are more evenly distributed throughout the specimen (Fig. 3). In contrast, the number of connections is the lowest amongst the three cores, although some pores have CN > 6 (Fig. 4c). These features may explain the highest *Ƭ* and the lowest *k* in BF35_Δ, despite its largest *n*_*eff*_.

##### Pore orientation and size ranking

**Fig. 5:**
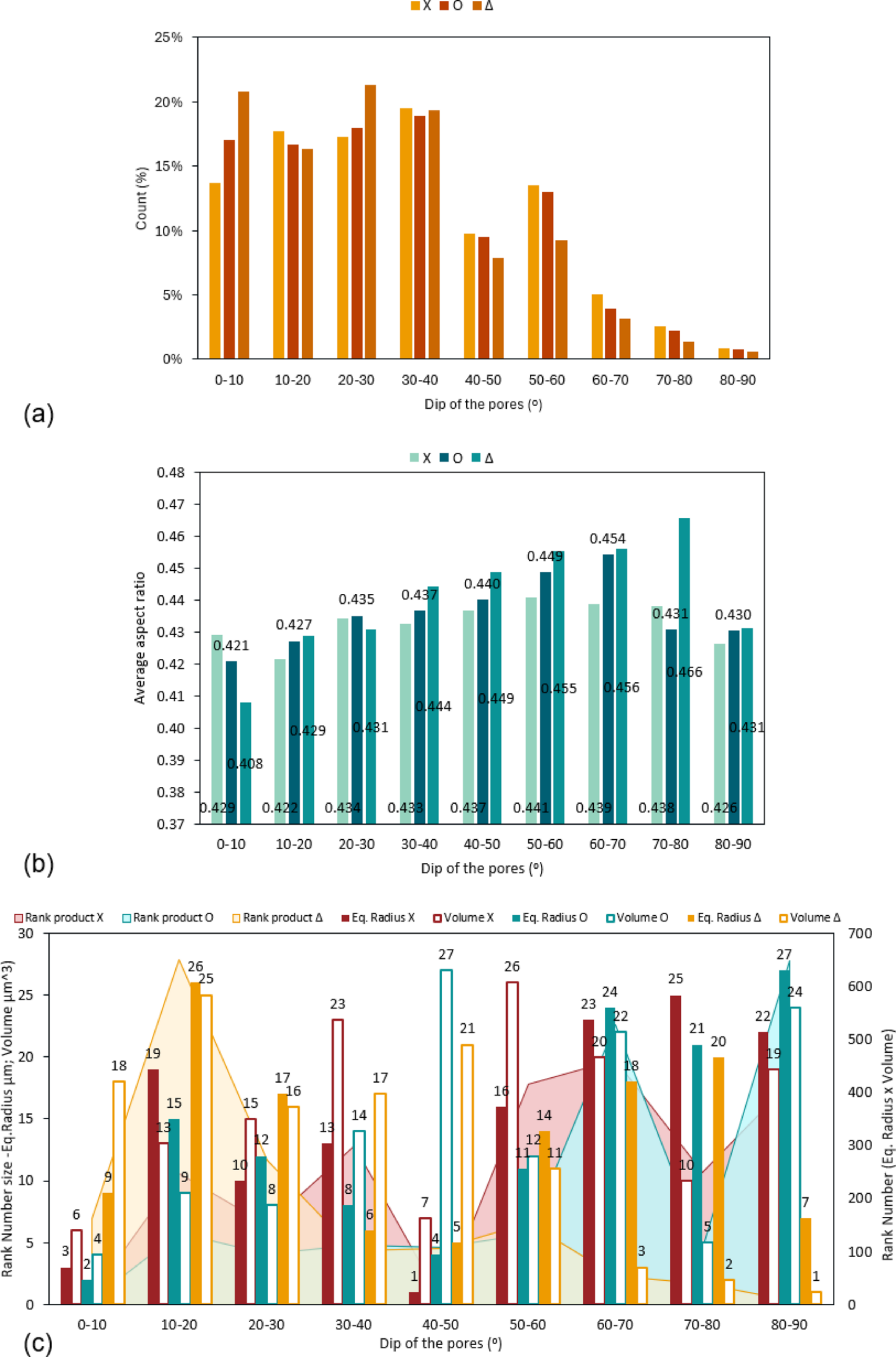
Variation of the number (a), mean aspect ratio (b), and rank systems for the mean equivalent radius, mean volume, and their product (c) for different inclinations/dip groups of the pores present in the cylinders produced along X, O, and Δ directions.

Visual classification of pores at the hand-sample scale across the three orthogonal directions can be misleading because it does not account for internal pores as µCT images. Therefore, pore dip angles were measured and grouped in 10° intervals (Fig. 5a).

Horizontal (perpendicular to the loading axis) pores (< 40°) are more prevalent than vertical (parallel to the loading axis) or diagonal ones (> 40°), contradicting macroscopic observations. For example, BF35_O contains both large, high-volume vertical pores (mean equivalent radius > 60 μm; mean volume > 9000 mm^3^) and numerous smaller horizontal pores (~ 27 μm; < 3000 mm^3^). Diagonal pores with significant average volume (22478 mm^3^) but smaller equivalent radius (24 μm) are also observed in BF35_O and other cores, highlighting the need to consider both the pore radius and volume as size metrics.

Average aspect ratios for each dip group are shown in Fig. 5b. The mean aspect ratio (0.437) is independent of direction and pore dip, with spheroidal pores predominating in the pore system.

The ranking system was applied to equivalent radius and pore volume, as they showed greater variability than aspect ratio. Pore count was excluded from the ranking due to its higher abundance in BF35_X. The ranks range from 1 (smallest mean radius) to 27 (largest), as shown in Fig. 5c. The combined pore size ranking system goes up to 650 (Fig. 5c).

As shown in Fig. 5c, pores with larger equivalent radii and volumes cluster within specific dip intervals: 70–80° and 50–60° for BF35_X, 80–90° and 40–50° for BF35_O, and 10–20° for BF35_Δ, respectively. The rank system reaches higher values for 60–70°, 80–90°, and 10–20° for BF35_X, BF35_O, and BF35_Δ, respectively.

Among these, BF35_O and BF35_X show the most heterogeneous pore size distribution relative to dip orientation at the microscale, supporting the trends observed in Fig. 4a. BF35_Δ contains predominantly horizontally aligned pores (perpendicular to the core axis) with higher mean volume, alongside with a significant number of vertically oriented ones (parallel to the core axis) with smaller volumes. In BF35_O, vertical pores dominate the pore space. Both BF35_X and BF35_O include several pores with dips > 60°, yet they produce the lowest and highest *σ*_*c_UCS*_, respectively (Table 1).

### Strength values and digital rock physics after failure

**Fig. 6:**
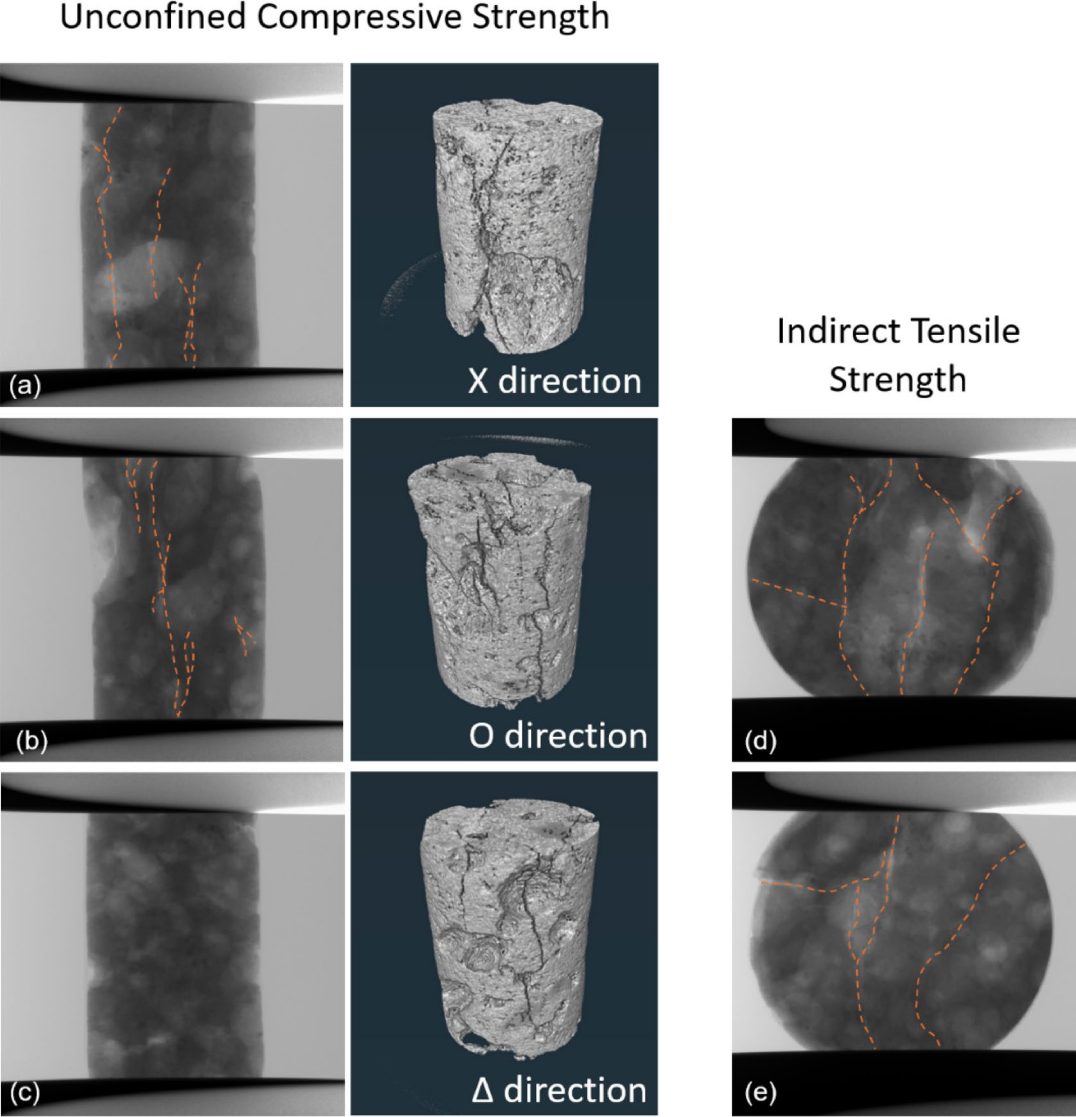
Mechanical test and post-deformation fracture pattern for BF35. Unconfined compressive strength test is represented by (a), (b), and (c) (cylinders cored from BF35.1), and indirect tensile strength was obtained via Brazilian test, represented in (d) and (e) (discs cored from BF35.2). All specimens fail by multiple tensile splitting. Deviations of the fracture pattern are induced by the larger pores. (d) Associates with the O direction, as most pores are parallel to the loading direction, while (e) translates from Δ direction, with most pores perpendicular to the loading direction.

All specimens show brittle behaviour during UCS and ITS tests (Fig. 6). Failure was characterised by tensile splitting, frequently involving the coalescence of cracks to form a dominant fracture. Crack propagation occurs primarily along the margins and tips of elongated pores, indicating these boundaries act as stress concentrators.

Although the Brazilian test imposes a tensile stress field that favours diametral splitting parallel to the loading axis, BF35 also exhibits pore-related crack patterns oriented perpendicular to the loading direction.

In UCS tests, both minor and major fractures develop parallel to the loading direction. *σ*_*c_UCS*_ decreases by approximately 50% (15 MPa) when elongated pores align parallel to the loading axis (O direction), whereas the other cylinders (direction X and Δ) show higher and comparable strength values (32–36 MPa).

The *σ*_*t*_ values are 4 MPa for BF35_O and 2 MPa for BF35_Δ, indicating that pores oriented perpendicular to the loading direction contribute to lower *σ*_*t*_.

BF35_X shows the highest strength and *n*_*T*_, despite having the lowest initial *n*_*eff*_ (Table 1). Its *n*_*eff*_ increases markedly after failure, suggesting that a high initial abundance of small pores combined with lower *Ƭ* and pore-scale heterogeneity may promote fracture-induced connectivity.

In contrast, BF35_O, characterised by the highest initial *n*_*eff*_, records the lowest strength but a substantial post-failure increase in *k*, indicating that high pre-deformation *n*_*eff*_ can contribute to enhanced post-deformation fluid connectivity. BF35_Δ shows minimal *k* increase, likely due to a lower density of pores and throats, and to large, horizontally aligned pores that contribute minimally to axial permeability.

### Physical and water transport properties

The physical and water transport properties at the hand-sample scale, including mean values and standard deviations, are summarised in Table 2. Following Benavente et al.^43^, anisotropy is defined as the ratio between the maximum and minimum values of *V*_*p*_, *V*_*s*_, *V*_*p*_*/V*_*s*_, *E*_*d*_, *υ*_*d*_, *n*_*cc*_, and *C*_*EN*_.

**Table 2:** Physical and water transport properties of BF35 six specimens along the different directions (O, X, Δ) and total mean values (avg) ± standard deviation (std.dv) of the several properties. *n*_*eff*_ – open porosity; *ρ* – dry density; *V*_*p*_*/V*_*s*_ – *P*-wave velocity (*V*_*p*_) to *S*-wave velocity (*V*_*s*_) ratio; *E*_*d*_ – dynamic Young’s modulus; *υ*_*d*_ – dynamic Poisson’s ratio; *C*_*EN*_ – capillary absorption coefficient; *n*_*cc*_ – capillary connected porosity; *A* – anisotropy, which is the ratio between the maximum and the minimum value.

BF35	V_*p*_ (m/s)	V_s_ (m/s)	V_*p*_/V_s_	E_d_ (GPa)	υ_d_	*n*_eff_ (%)	ρ (g/cm^3^)	*n*_cc_ (%)	C_EN_ (g/m^2^ s^0.5)^
1_X	2977	1972	1.51	19.75	0.11	19.63	2.290	5.01	3.04
1_O	2993	1969	1.52	19.86	0.12			5.11	3.00
1_Δ	2895	1469	1.97	13.11	0.33			5.03	2.94
A	0.97	0.74	0.77	0.66	0.33			0.98	0.97
2_X	3065	1445	2.12	13.02	0.36	19.78	2.299	4.70	2.80
2_O	3094	1827	1.69	18.91	0.23			4.65	2.76
2_Δ	2951	1750	1.69	17.30	0.23			4.65	2.74
A	0.95	0.79	0.79	0.69	0.64			0.99	0.98
3_X	2961	1336	2.22	11.17	0.37	20.12	2.280	4.93	2.92
3_O	3012	1401	2.15	12.19	0.36			4.85	2.86
3_Δ	2817	1507	1.87	13.45	0.30			4.92	2.90
A	0.94	0.89	0.84	0.83	0.80			0.98	0.98
4_X	3005	1641	1.83	15.68	0.29	20.18	2.262	5.11	3.04
4_O	3032	1842	1.65	18.53	0.21			5.16	3.03
4_Δ	2802	1703	1.64	15.84	0.21			5.24	3.08
A	0.92	0.89	0.90	0.85	0.72			0.98	0.99
5_X	3025	1782	1.70	18.22	0.23	18.51	2.325	5.58	3.17
5_O	3076	1935	1.59	20.41	0.17			5.86	3.35
5_Δ	2855	2014	1.42	18.95	-			5.39	3.10
A	0.93	0.88	0.83	0.89	-			0.92	0.93
6_X	3208	2013	1.59	21.74	0.18	19.87	2.283	5.23	2.99
6_O	3126	1498	2.09	13.84	0.35			5.05	2.85
6_Δ	2981	2043	1.46	20.14	-			5.24	3.00
A	0.93	0.73	0.70	0.64	0.16			0.96	0.95
Avg± std.dv	2993 ± 102.29	1730 ± 231.72	1.76 ± 0.25	16.78 ± 3.29	0.23 ± 0.11	19.68 ± 0.57	2.290 ± 0.020	5.10 ± 0.31	2.98 ± 0.15

BF35 specimens have *n*_*eff*_ ranging from 18.51 to 20.18%, with *ρ* varying between 2.262 and 2.325 g/cm^3^. An inverse correlation is observed between these parameters, where specimens with the lowest *n*_*eff*_ display the highest *ρ*.

*n*_*eff*_ values in Table 2 align closely with pre-deformation data reported in Table 1 indicating that the selected nominal voxel size was appropriate for representing the pore space system of BF35 samples.

In addition, *n*_*eff_Hg*_ represents 65% of the total mean *n*_*eff*_, reflecting the limited measurable pore size range of MIP (up to 180 μm), which does not capture the large pores identified by µCT. Moreover, large pore bodies may be connected by narrower throats (ink-bottle effect), leading to an underestimation of connected porosity, as mercury intrusion is dictated by throat size.

An incipient trend was observed in the *P*-wave velocity of BF35, with *V*_*p*_ being higher in the O direction and lower in the Δ direction. Despite this trend, the *V*_*p*_ values (2802–3208 m/s) are generally consistent across specimens and orientations, yielding anisotropy values exceeding 0.90. In contrast, *V*_*s*_ exhibits greater directional variability (1445 to 2043 m/s), thus related to a higher standard deviation than *V*_*p*_. Consequently, *E*_*d*_ and *υ*_*d*_ may show anomalous trends and more pronounced anisotropy (Table 2).

The *n*_*cc*_ (4.65–5.86%) represents, on average, 25% of *n*_*eff*_, reflecting the proportion of connected pores that actively participate in the capillary water absorption.

**Fig. 7:**
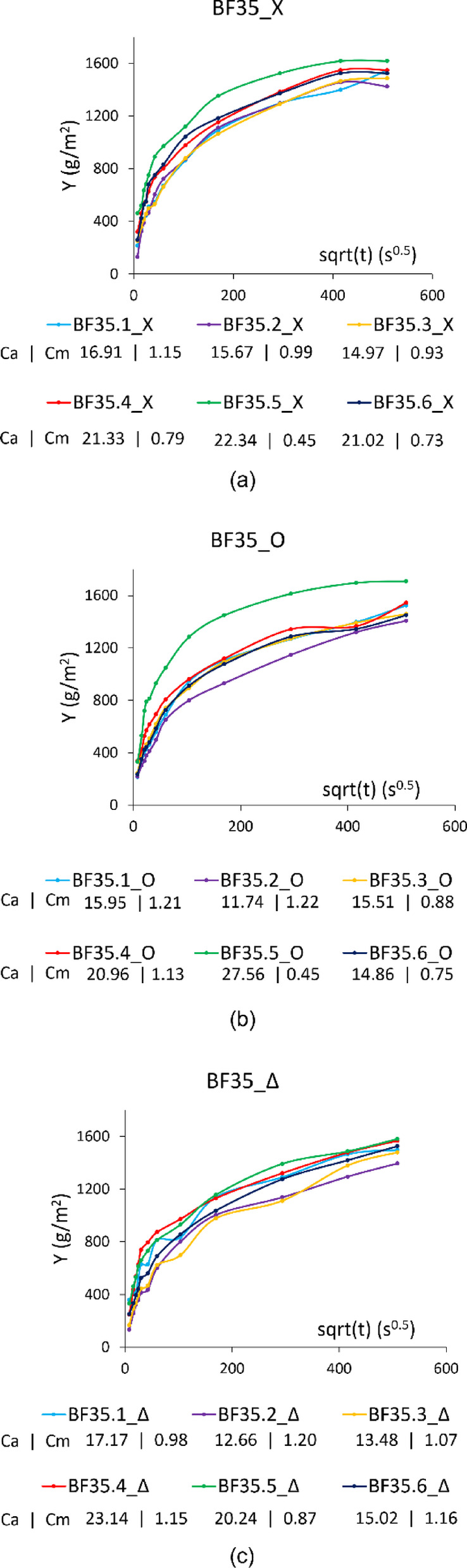
Water absorption curves along t^1/2^ for BF35_X; (b) BF35_O; (c) BF35_Δ. *Y* is the water absorption. All specimens exhibit nonlinear behaviour, and, therefore, intermediate anomalous (*C*_*a*_) and late (*C*_*m*_) absorption coefficients, in g/m^2^·s^0.5^, were considered.

Capillary parameters do not follow any specific trend with direction as *V*_*p*_, with higher absorption occurring for any of the three directions. Thus, *n*_*cc*_ and *C*_*EN*_ (2.74–3.35 g/m²·s⁰^·^⁵) are consistent across directions and specimens, leading to anisotropy values higher than 0.90, which indicate a homogenous water uptake, as also illustrated by the water absorption curves (Fig. 7). Within 24 h, BF35 specimens, which have a nonlinear water absorption, reach a plateau that indicates a near-saturation state. In addition, *C*_*e*_ (*C*_*m*_ + *C*_*a*_) is consistently higher than *C*_*EN*_ because the latter does not account for the different absorption stages like the SF model but rather considers the total water uptake over the test duration, approximating from *C*_*m*_ values.

The absence of directional heterogeneity at the hand-sample scale in BF35 cubic specimens is, therefore, not reflected by *C* or *V*_*p*_*/V*_*s*_ despite the localised variations in porosity and strength observed in the µCT sub-specimens. Such an aspect is further addressed in the following section.

## Discussion and conclusions

Extensive observations in Azorean lavas^3^ ranging from basalts to trachytes and textures from dense to vesicular, with varying degrees of fracturing and pore size distributions, have underscored the critical role of heterogeneities in volcanic rocks. Such materials typically comprise a complex assemblage of spherical-to-spheroidal pores, which may also include microcracks (e.g., penny-shaped fractures) in varying proportions^3,44^. Within this framework, the present study systematically examines how heterogeneities influence rock properties through multiple complementary methods that enable their 3D multiscale characterisation.

The results demonstrate that significant variations in pore-space architecture, and thus in transport and mechanical properties, occur as a function of lava orientation.

PSD from MIP analysis of BF35 lava shows a bimodal distribution, whereas the digital rock analysis reveals large coalescent pores, suggesting prolonged bubble evolution during magma ascent and lava emplacement.

Regarding pore shape, BF35 has more elongated pores (lower aspect ratio) than, for example, the andesites from Volcán de Colima^45^, which were microstructurally assessed for pore geometry characteristics, besides sharing a connected porosity (7.9–25.0%) and mineralogy similar to BF35, serving as a direct benchmark.

Although a preferred orientation is visible in BF35 hand-specimens, 3D microanalysis reveals pores distributed across multiple dip angles, which mitigates the weakening effect of directional elongation. As such, the complexity of the pore space limits the ability to isolate the impact of pore shape on the physical and mechanical properties. Consequently, numerical models, such as those proposed in previous works^4,6,9^, remain essential for understanding these mechanisms.

The introduction of innovative 3D non-destructive digital rock physics investigations greatly facilitates the study of the complex heterogeneities in natural rocks. When combined with in situ mechanical tests, these methods allow researchers to establish direct links between macroscopic properties and microstructural features. In particular, the conducted time-resolved tests elucidated the mechanical behaviour and highlighted how pore geometry and spatial distribution influence crack development. These microstructural factors are consistent with the observed directional variations in strength.

For example, in UCS tests, both minor and major fractures develop parallel to the loading direction, consistent with microstructural^45^ and macroscopic observations^3^ in similar rocks.

The weakening observed in BF35_O (i.e., parallel to the preferred orientation of pores) is attributed to large diagonal (40–50°; Fig. 5c) and vertical pores (80–90°; Fig. 5c) extending nearly half the specimen length and positioned near the edge (Fig. 6b). Large pores of comparable size but different orientation occur in BF35_X (50–60°; Fig. 5c) and BF35_Δ (10–20°; Fig. 5c), which show similar strengths (32–36 MPa). This comparison suggests that, more generally, the spatial distribution of pores (rather than orientation alone) ultimately exert a crucial control on strength.

Such pores favour stress concentration, microcracking at lower stress levels, and fracture propagation, indicating that *σ*_*c_UCS*_ can be strongly affected by the spatial distribution of large pores along the specimen length. This observation supports numerical models showing that larger, closely spaced, and non-uniformly sized pores can reduce rock strength^4,46^.

In the Brazilian tests, crack patterns deviated from the standard central or central-multiple types described by Basu et al.^47^, often propagating perpendicular to the loading direction due to the specific pore architecture of BF35. These secondary cracks reflect local stress reorientation around pores, which locally modifies the principal stress directions imposed by the test. Although the dataset is limited (4 MPa for BF35_O and 2 MPa for BF35_Δ), the results suggest that pores oriented perpendicular to the loading direction reduce *σ*_*t*_, consistent with the observations by Heap et al.^48^.

In addition, mechanical testing on ~ 8 mm diameter cylinders and ~ 13 mm diameter discs suggests that strength is relatively scale-independent in intact samples when the L/D ratio remains constant, in agreement with findings by Van Stappen et al.^49^ for limestones. However, at the rock mass and volcanic edifice scale, discontinuities may reduce the overall stiffness and strength compared with laboratory specimens^50^.

Regarding *k* simulations (Table 1), pre-deformed lavas tend to be poorly permeable, but brittle failure can significantly increase the permeability of BF35, particularly when the pore network is dense, well connected, and distributed throughout the specimen. These results help clarify how porosity influences permeability and its evolution during failure, with implications for volcanic processes such as outgassing^51^ and hydrothermal fluid migration^52,53^.

The PNM also indicate that throat length is not the primary control on permeability. Instead, permeability is more influenced by the distribution of throat radii and pore connectivity. Moving towards the Δ direction (i.e., perpendicular to the preferred orientation of pores), the samples display narrower pore and throat size distributions and fewer connections in the PNM. These characteristics result in higher tortuosity and lower permeability despite relatively high connected porosity.

It is important to note that throats with equivalent radii > 10 μm can act as sinks for supersaturated solutions, promoting processes such as salt crystallisation without necessarily inducing stress damage^13^. In fact, the PSD controls water transport mechanisms and, consequently, the behaviour of rocks exposed to deleterious agents.

In summary, MIP and digital rock analyses indicate that:

- Mechanical behaviour, particularly UCS, is influenced by the size and spatial distribution of pores relative to the specimen length, especially through the presence of large pores. Variations in pore dip angles within the natural samples hinder the influence of directional pore elongation on strength.
- 3D microstructural analysis proved essential, providing PNM that offer insights beyond visual inspection and conventional porosimetry.

Although pore architecture and strength of BF35 vary with orientation, the capillary water absorption and *V*_*p*_*/V*_*s*_ ratio remain similar across all directions.

Although directional capillary effects are suppressed at the hand-sample scale due to large pores (> 1 mm), PNM indicate that capillary flow is primarily governed by pore connectivity and throat size distribution at the pore scale. For instance, BF35_X shows a more heterogeneous pore structure with numerous small pores (< 101 μm) and a broad throat radius distribution (Fig. 4d), combined with a higher CN, which promotes connectivity (and permeability) despite having the lowest *n*_*eff*_ among the three cores.

In contrast, BF35_O and BF35_Δ exhibit a more open structure dominated by pores connected by throats with relatively uniform radii and greater lengths (Fig. 4d), resulting in similar larger connected porosity (and lower permeability) and broadly comparable pore-network geometries (Fig. 3). Despite these microscale differences, capillary absorption appears only weakly influenced by pore orientation at the hand-sample scale, leading to comparable *C* values. A similar behaviour is observed for ultrasonic wave velocities, likely reflecting the comparable bulk porosity.

Vairé et al.^6^ further argue that preferred pore shape orientation should be considered when interpreting seismic data, and low aspect ratios have been found to produce relevant *V*_*p*_*/V*_*s*_ variations in trachyandesites^5^. However, in this study, the impact of pore elongation on dynamic properties remains inconclusive. Although *V*_*p*_ showed an incipient directional trend, the variability in *V*_*s*_ likely reflects the complex 3D microstructure.

According to the classification of Graue et al.^54^, BF35 has low or moderate capillary absorption capacity, depending on whether *C*_*EN*_ or *C*_*m*_ + *C*_*a*_ (SF model) is considered. Previous studies^34,35^ have determined that bimodal to polymodal PSD, as in BF35, are better explained by the SF model because it effectively captures the various stages of water absorption than the standard approach.

The moderate capacity of BF35 to absorb water by capillarity is attributed to the presence of large pores, many exceeding 1 mm in radius, which lie above the upper limit for capillary-active pores (100 μm^43^);. These findings are consistent with observations for other high-porosity rocks, including basalts^21^ and sedimentary lithologies^13^.

According to Jurin’s Law, capillary rise is inversely proportional to pore radius^36^. For pores with a radius of 1 mm, the theoretical capillary rise is approximately 1.5 cm (assuming water at 25 °C), confirming that gravity-driven flow dominates water transport in BF35, relative to the specimen height (~ 30 mm). As a result, permeability appears to be the predominant water transport mechanism in BF35.

Capillary imbibition is one of the most important parameters in the degradation of building stones^15,16^ and the dominant water transport mechanism in rocks exposed to the atmosphere^14^, such as on volcanic slopes. Although minor, vapour adsorption and capillary condensation may also operate (Fig. 2), either accelerating or reducing the resistance to crack growth, as previously reported^55–57^. These mechanisms are associated with time-dependent fracturing^58^, known to increase seismic activity preceding volcanic eruptions^59^.

In brief, non-destructive techniques applied to BF35 hand-samples show:

- No consistent trends in ultrasonic wave velocities, dynamic moduli, and capillary absorption values across distinct directions.
- A moderate capillary water absorption that is better described by SF model. However, gravitational flow predominates in BF35 due to the presence of pores larger than 1 mm.

In conclusion, this research shows that 3D pore-scale heterogeneity in vesicular lavas, such as BF35 (S. Miguel, Azores, Portugal), can produce directional variations in porosity and strength at the microscale, highlighting the role of the pore-scale structure. In contrast, other properties, including ultrasonic wave velocities and capillary absorption coefficient, may remain unaffected across directions at the hand-sample scale.

This study demonstrates the advantage of a multiscale approach combining pore-scale imaging, MIP, and bulk physical measurements to capture the heterogeneity that single techniques cannot fully resolve. Such integration provides clearer insight into of how pore structure controls water transport and mechanical properties of vesicular lavas.

For the future development of empirical equations linking pore structure and mechanical strength, datasets should include a larger number of specimens and lithologies with a wider range of porosities and systematically measured pore and throat metrics, capillary absorption, and mechanical properties. These empirical equations will not only enable more robust generalisation across lithologies and scales but also provide practical tools for engineering applications in volcanic terrains.

## Data Availability

The main data generated and analysed during this study are included in this published article. Further data can be provided from the corresponding author on reasonable request.

## Declarations

### Permissions and sampling authorization

Lava samples were collected exclusively from non-protected areas. Under the applicable regional legislation of the Azores (Decreto Regulamentar Regional n.º 20/2012/A, de 5 de novembro; Decreto Legislativo Regional n.º 10/2019/A, de 22 de maio), no permit or prior authorization is required for geological sampling outside legally protected areas.
